# Circulating tumor DNA predicts prognosis at different time points in patients with esophageal cancer: a systematic review and meta-analysis

**DOI:** 10.3389/fonc.2025.1608872

**Published:** 2025-09-25

**Authors:** Min Wang, Chunbin Xiong, Siyu Wang, Yu Qiu, Zongqi Hou, Peng Gao

**Affiliations:** ^1^ Department of Laboratory, The First People’s Hospital of Yibin, Yibin, China; ^2^ Department of Oncology, The First People’s Hospital of Yibin, Yibin, China

**Keywords:** esophageal cancer, liquid biopsy, circulating tumor DNA, prognostic biomarker, systematic review and meta-analysis, tumor-informed assay

## Abstract

**Background:**

Circulating tumor DNA (ctDNA), as a liquid biopsy biomarker, is undergoing extensive evaluation for its clinical utility across multiple tumor management scenarios. This study aimed to assess the prognostic value of ctDNA in esophageal cancer (EC) patients at different treatment time points through a systematic review and meta-analysis.

**Materials and methods:**

A comprehensive search of the PubMed, Embase, and Cochrane Library databases from construction to October 2024 was conducted, and studies investigating the association between ctDNA and progression-free survival (PFS) and overall survival (OS) in EC patients were screened for inclusion. Primary outcomes included PFS/OS by ctDNA status at different time points (baseline, after neoadjuvant therapy, and during follow-up). Risk ratios (HRs) for PFS/OS with positive ctDNA tests at various time points were combined, and subgroup analyses were conducted for tumor-informed and non-tumor-informed testing of ctDNA.

**Results:**

A total of 22 studies involving 1519 patients were finally included in this meta-analysis. In univariate analyses, detection of ctDNA was associated with poorer PFS at baseline (HR = 1.64, 95% CI:1.30-2.07), after neoadjuvant therapy (HR = 3.97, 95% CI: 2.68-5.88) and during follow-up (HR = 5.42, 95% CI:3.97-7.38). Similarly, detection of ctDNA at all time points was associated with poorer OS (at baseline: HR = 2.02, 95% CI:1.36-2.99; after neoadjuvant therapy: HR = 3.41, 95% CI: 2.08-5.59; and during follow-up: HR = 4.93, 95% CI:3.31-7.34). Similar PFS and OS outcomes were observed in multivariate analyses. The uni- and multivariate combined HRs for PFS/OS with ctDNA detected at different time points were numerically related as: baseline < after neoadjuvant therapy < during follow-up(PFS:1.90→4.07→5.22; OS:2.39→3.15→5.37). When ctDNA was detected in combined tumor-informed assays at baseline and after neoadjuvant therapy, most HRs for recurrence and mortality risk showed a trend toward higher values compared with non-tumor-informed assays. ctDNA test positivity predicted clinical recurrence an average of 4.53 months earlier (range: 0.98-11.6 months) than conventional radiological imaging techniques.

**Conclusions:**

Positive ctDNA testing was associated with poorer prognosis throughout the treatment period of EC patients, and the prognostic value of monitoring ctDNA status increased with time from baseline to follow-up.

**Systematic Review Registration:**

https://www.crd.york.ac.uk/PROSPERO/view/CRD42024612909, identifier CRD42024612909.

## 1 Introduction

According to the Global Cancer Observatory 2022, esophageal cancer (EC) is the 11th most common cancer and the 7th leading cause of cancer deaths globally, resulting in about 510,000 new cases and about 450,000 deaths (1).EC mainly consists of esophageal squamous cell carcinoma (ESCC) and esophageal adenocarcinoma (EAC). EC patients are asymptomatic or atypical in the early stages. Because of their very aggressive biology it often leads to a poor prognosis due to significant progression of the disease by the time of diagnosis. In most countries and regions, the 5-year survival rate after EC diagnosis is between 10% and 30% (2). Currently, the treatment of EC is based on a staged combination model, in which patients with locally advanced disease are usually treated radically with a multimodal approach including surgery, e.g., neoadjuvant therapy in combination with surgery, and palliatively with advanced (metastatic or disseminated) and recurrent disease (3). Although the treatment modalities of EC have improved significantly, the clinical management of EC is still unable to accurately and timely capture the recurrence risk of patients under the current clinical technology, resulting in no significant improvement in their prognosis. There is an urgent need to develop a new biomarker to predict the risk of EC patients at different stages of treatment and to implement individualized treatment so that timely intervention can be made to improve overall survival outcomes.

In recent years, more and more researchers have begun to focus on applying liquid biopsy in oncology, among which circulating tumor DNA (ctDNA) has attracted much attention (4). ctDNA is released into the bloodstream during apoptosis or necrosis of tumors and through circulating tumor cells (5–8). Compared with clinically available serum tumor biomarkers (e.g., CEA, SCC), ctDNA has higher sensitivity and specificity (8, 9). ctDNA has a very short half-life of approximately 2 hours, which allows it to reflect the real-time tumor load of a tumor patient more accurately and to be used as a dynamic biomarker for tracking the presence of tumors (10, 11). Some studies have reported the application of ctDNA in the early diagnosis of tumors, identification of tiny residual lesions, response to treatment, and survival prognosis, respectively (12–15). Most studies focused on the relationship between ctDNA and survival prognosis in EC. There are three systematic reviews and meta-analyses on the relationship between ctDNA and EC prognosis (16–18), the most comprehensive of which is from Zhang and Jin et al. (18). They conducted a complete subgroup analysis of the prognosis of ctDNA in patients with EC in terms of different ethnicities, histological subtypes, anatomical locations, ctDNA testing methods, testing times, and different treatment modalities. Although previous studies have confirmed the prognostic value of ctDNA in EC, a core issue that has been overlooked is that the vast majority of research has pooled ctDNA data collected at different time points for analysis, failing to adequately account for the inherent dynamic evolution of ctDNA levels over time and in response to treatment. This ‘static’ analytical approach fails to capture the full value of ctDNA as a biomarker that reflects tumor burden and treatment response in real time. This study aims to systematically evaluate the prognostic value of ctDNA at three critical clinical time points (baseline, after neoadjuvant therapy, and follow-up) using a “dynamic” framework. It further compares the differences in clinical recurrence detection timing between ctDNA and traditional imaging techniques, seeking to provide precise evidence for the application of ctDNA at specific clinical decision points.

## 2 Materials and methods

The present systematic review and meta-analysis were conducted by the Preferred Reporting Items for Systematic Reviews and Meta-Analyses (PRISMA) (**Supplementary Table S1**) and the Assessing Methodological Quality in Systematic Reviews (AMSTAR) guidelines (19, 20). The study protocol was registered on the International Prospective Register of Systematic Reviews (PROSPERO) (ID: CRD42024612909).

### 2.1 Literature retrieval

We searched PubMed, Embase, and the Cochrane Library for studies published from inception to 23 October 2024, with no language restriction. The search strategy was for studies related to “esophageal neoplasms” and “circulating tumor DNA,” and the search formula took the form of a combination of subject terms plus free words/synonyms, with the complete search string shown in the **Supplementary Table S2**. All abstracts identified by the search strategy were screened first, followed by a review of potentially eligible full texts, and eligible conference abstracts were also included in the study. Two authors(MW and CX) independently performed study screening, data extraction, and quality assessment. Any disagreements were resolved through discussion with the other author(SW).

### 2.2 Study selection

Inclusion criteria: (a) clinical studies (prospective or retrospective); (b) patients with pathologically confirmed primary EC, regardless of the specific treatment modality; we classified adenocarcinoma of the esophagogastric junction as EAC due to its similarity to EAC in terms of clinical features and treatment strategy; (c) plasma samples with measurable ctDNA; there were no limitations on the methods of detection and analysis of ctDNA, considering the lack of a gold standard and the lack of direct comparisons between the different methods; and (d) provide data on the association between ctDNA and outcome [progression-free survival (PFS) and overall survival (OS)].

Exclusion criteria: (a) case reports, comments, letters, reviews, and systematic reviews and meta-analyses; (b) non-human studies; (c) replicated studies; (d) no relevant data.

### 2.3 Data extraction

Data to be extracted include (a) general information (title, authors, date of publication and type of study);(b) Patient population characteristics (sample size, tissue subtype, clinical stage and treatment modality); (c) The timing of ctDNA testing of plasma samples, the method of testing, and whether the test is tumor-informed and significantly mutated genes;(d) Duration of follow-up and advance time to ctDNA detection of disease recurrence;(e) Hazard ratios (HRs) and 95% confidence intervals (CIs) for PFS and OS. If survival data were not provided directly in the text, Engauge Digitizer version 11.1 could be used to extract non-numerical survival data from Kaplan-Meier curves if necessary, and HRs and 95% CIs could be calculated using Tierney’s method (21).

### 2.4 Quality assessment

The risk of bias for included studies was assessed using the Newcastle-Ottawa Scale (NOS) instrument. This instrument consists of three components: selection, comparability, and outcome, with studies scored from 0 to 9 (22). Scores of 0-3, 4-6, and 7–9 were defined as low, moderate, and high-quality studies, respectively.

### 2.5 Objectives and outcomes

The different time points for ctDNA assessment are classified as baseline (after diagnosis and before any treatment), after neoadjuvant therapy (after neoadjuvant therapy and before surgery), and during follow-up (during adjuvant therapy or follow-up). ctDNA testing can be either tumor-informed or non-tumor-informed, with tumor-informed assays defined as ctDNA assessments based on initial genomic analysis of primary tumor tissue samples to identify tumor-derived alterations. In contrast, non-tumor-informed assays do not require concomitant tumor tissue sequencing (9, 23).

The main objective was to assess whether patients with positive ctDNA tests in plasma samples at different time points had a worse prognosis (PFS and OS) than patients with negative ctDNA tests. The definitions of PFS and OS were based on the studies. PFS includes any recurrence events, such as disease-free survival (DFS), recurrence-free survival (RFS), freedom from progression (FFP), and time to progression (TTP); OS includes any type of mortality event, such as disease-specific survival (DSS) and cancer-specific death (CSD). The secondary objective was to perform subgroup analyses of ctDNA tumor-informed or non-tumor-informed testing at different time points. Finally, the time to detection of clinical recurrence by ctDNA was compared with conventional imaging techniques.

### 2.6 Statistical analysis

ctDNA was considered a binary (positive/negative) variable for the analysis. Univariate and multivariate HRs and 95% CIs were extracted, if available, and then combined separately. Multivariate HRs were extracted irrespective of the variables included in the model for each study. HRs greater than 1 indicated that patients with EC who tested positive for ctDNA had a poor prognosis. The I^2^ statistic was used to assess statistical heterogeneity between studies. If statistical homogeneity was observed, a fixed-effects model was used to combine the results (P > 0.05 or I^2^ < 50%), whereas a random-effects model was used if statistical heterogeneity existed (P < 0.05 or I^2^ > 50%). If a high degree of heterogeneity existed, sensitivity analyses were performed by systematically excluding individual studies to assess the stability of the results. In addition, publication bias was examined for results containing 10 or more studies using visual assessment of funnel plots. All analyses were performed using RevMan 5.3 software (Cochrane, London, UK).

## 3 Results

### 3.1 Search results and essential characteristics

From the 765 records identified, this systematic review and meta-analysis finally screened for the inclusion of 22 studies (involving 1519 patients) (24–45), including 20 full-length articles and two conference abstracts (31, 43). **Supplementary Figure S1** shows a flowchart of the study selection process according to PRISMA. The primary clinical characteristics included in the studies are shown in **Table 1**. Of the included studies, 11 were prospective, and 11 were retrospective. 9 studies focused on EAC, eight on ESCC, and the remaining five on EAC+ESCC. Based on TNM staging, it was estimated that approximately 74.0% of the study population were in stage 0-III, and 26.0% were in stage IV. These studies included the following treatment modalities: (1) curative surgery (24, 28, 30, 34, 37, 39–41); (2) neoadjuvant therapy followed by surgery (25, 27–29, 31, 32, 34–38, 40, 42); (3) definitive radiotherapy/radiochemotherapy (26, 32, 36, 40, 42); and (4) palliative chemotherapy (28, 33, 40). Follow-up time ranges from 24 to 100 months.

**Table 1:** Clinical characteristics of included studies.

Name	Study design	Sample size	Main subtype	Clinical stage	Treatment	FUP(Median)/months
Li 2024 (24)	prospective	17	ESCC	I-III	surgery	24(NR)
Iden 2024 (25)	prospective	86	EAC	III-IV	NAT + surgery	36(26.7)
Chen 2024 (26)	prospective	42	ESCC	I-IVA	definitive CRT	36(27.6)
Van den ende 2023 (27)	prospective	111	EAC	II-III	NAT + surgery	80(NR)
Ng 2023a (28)	prospective	74	ESCC	I-IV	surgery NAT + surgery	53(NR)
Ng 2023b (28)	prospective	73	ESCC	I-IV	palliative CT	40(NR)
Morimoto 2023 (29)	prospective	16	ESCC	0-IV	NAT + surgery	32(NR)
Liu 2023 (30)	prospective	57	ESCC	0-III	surgery	>62(40.87)
Lander 2023 (31)	prospective	42	EAC+ESCC	I-III	NAT + surgery	30(22.3)
Wang 2022 (32)	prospective	40	ESCC	II-IVB	NAT + surgery definitive RT/CRT	36(20.6)
Van velzen 2022 (33)	retrospective	63	EAC+ESCC	IV	palliative CT	67(NR)
Huffman 2022 (34)	retrospective	212	EAC+ESCC	I-III	surgery NAT +surgery	83(13.9)
Hofste 2022 (35)	retrospective	78	EAC+ESCC	IB-IIIB	NAT + surgery	>55(NR)
Cabalag 2022 (36)	retrospective	62	EAC	I-IV	NAT + surgery definitive CRT	100(15.2)
Bonazzi 2022 (37)	retrospective	57	EAC	I-III	surgery NAT + surgery	100 (37)
Ococks 2021 (38)	prospective	97	EAC	II-IV	NAT + surgery	80(32.9)
Liu 2021 (39)	retrospective	53	ESCC	0-III	surgery	60(34.8)
Iwaya 2021 (40)	retrospective	36	ESCC	I-IV	surgery NAT + surgery definitive CRT palliative CT	48(13.1)
Openshaw 2020 (41)	prospective	23	EAC	I-III	surgery	42(NR)
Azad 2020 (42)	retrospective	45	EAC+ESCC	IA -IIIB	NAT + surgery definitive CRT	67(NR)
Mohamed 2019 (43)	retrospective	36	EAC	I-IV	NR	NR(NR)
Maron 2019 (44)	retrospective	144	EAC	IV	NR	40(NR)
Kato 2018 (45)	retrospective	55	EAC	II-IV	NR	28.5(NR)

NR, not reported; NAT, Neoadjuvant therapy; CRT, chemoradiotherapy; CT, chemotherapy; RT, radiotherapy; FUP(Median), follow-up time (median follow-up time).

Specific information related to ctDNA in the included studies is presented in **Table 2**. Detection of ctDNA Two main methods were applied: (1) next-generation sequencing (NGS)-based assays, including TA-Seq and CAPP-Seq. (2) digital PCR (dPCR)–based assays, including droplet digital PCR (ddPCR). Most studies (18/22) had tumor-informed ctDNA assays (24, 26, 28–31, 33–37, 39–45). These 18 studies reported the genetic mutations detected in tumor tissue and/or plasma ctDNA.TP53 was the most commonly detected mutation, either in ESCC or EAC. Most TP53 variants are either missense or nonsense mutations. Other frequently mutated genes include NFE2L2, CDKN2A, PIK3CA, NOTCH1, ERBB2, ARID1A and SMAD4. Notably, most of the highly prevalent mutated genes are tumor suppressor genes. These gene mutations have been partially shown to be associated with poor prognosis in EC patients. The specific definitions of ctDNA-positive/negative status vary across studies. Most studies directly categorize patients into ctDNA-detectable and ctDNA-undetectable groups. At the same time, a minority employ classification methods that include variant allele frequency (VAF) thresholds combined with tumor fraction levels, mutation burden levels, or the presence/absence of specific genetic alterations.

**Table 2:** Information related to ctDNA in the included studies.

Name	Detection method	Tumor-informed assay	Mutational landscape	ctDNA(positive vs negative)
Li 2024 (24)	NGS	Yes	NR	ctDNA+ vs ctDNA-
Iden 2024 (25)	ddPCR	No	NR	ctDNA+ vs ctDNA-
Chen 2024 (26)	NGS	Yes	TP53 (68%), CDKN2A (20%), NFE2L2 (15%) , LRP1B (13%), NOTCH1(10%)	ctDNA+ vs ctDNA-
Van den ende 2023 (27)	NGS	No	TP53 (72%) , KRAS (13%), CDKN2A (11%)	ctDNA (VAF > 1% or ichorCNA > 3% vs ≤ 1% or ≤ 3%) ctDNA (non-clearance or ichorCNA > 3% vs clearance or ≤ 3%)
Ng 2023a (28)	Sanger sequencing + dPCR	Yes	TP53(51.3%) , NFE2L2 (13.5%) , PIK3CA(6.8%)	ctDNA(>1 Alterations vs ≤1 Alteration) ctDNA (alterations presence vs absence)
Ng 2023b (28)	Sanger sequencing + dPCR	Yes	P53(64.4%) , NFE2L2(21.9%) , PIK3CA(13.7%)	ctDNA NEF2L2 mutation (Yes vs No)
Morimoto 2023 (29)	NGS	Yes	TP53(85%), CDKN2A (8%), NFE2L2 (8%)	ctDNA+ vs ctDNA-
Liu 2023 (30)	NGS	Yes	TP53 (59.6%) , PTCH1 (35.1%) , PIK3CA (31.6%) , EGFR (21.1%), ATM(17.5%), PDGFRA(17.5%), MET(15.8%), SMAD4(15.8%), ALK(15.8%), BRAF (12.3%), ERBB2(10.5%), PTEN(10.5%)	ctDNA TP53 mutation (Yes vs No)
Lander 2023 (31)	NGS	Yes	NR	ctDNA+ vs ctDNA-
Wang 2022 (32)	NGS	No	TP53 (85.7%) , PRSS3 (21.4%) , CDKN2A (17.9%) , ART (14.3%), PIK3CA (14.3%), NFE2L2(11%), NOTCH1(11%), TGFB1(11%)	ctDNA+ vs ctDNA-
Van velzen 2022 (33)	NGS	Yes	TP53 (60%) , KRAS (22%), CDKN2A (7%)	ctDNA(≥ 2 mutations vs 0 or 1) ctDNA+(1 or more mutations with a VAF≥ 1%) vs ctDNA-
Huffman 2022 (34)	NGS	Yes	TP53 (94%), CDKN2A(13%), MSI(12%), SMAD4(10%), KRAS(9%)	ctDNA+ vs ctDNA-
Hofste 2022 (35)	NGS	Yes	TP53 (80%), SMAD4(42%), CDKN2A(27.4%), ARID1A(19.4%), APC(11.3%), KRAS(11.3%), PIK3CA(9.7%)	ctDNA+ vs ctDNA-
Cabalag 2022 (36)	ddPCR+NGS(TA-Seq)	Yes	TP53 (80%), SMAD4(42%), CDKN2A(27.4%), ARID1A(19.4%), APC(11.3%), KRAS(11.3%), PIK3CA(9.7%)	ctDNA+ vs ctDNA-
Bonazzi 2022 (37)	NGS	Both	TP53(46%)	ctDNA[Shedder vs (No variant detected and Non-shedder]
Ococks 2021 (38)	NGS	No	TP53 (15%), APC (8%) , KRAS (6%)	ctDNA+ vs ctDNA-
Liu 2021 (39)	NGS	Yes	TP53 (58.49%) , PIK3CA (16.98%) , EGFR (7.55%) , PTCH1 (5.66%)	ctDNA+ vs ctDNA-
Iwaya 2021 (40)	NGS+dPCR	Yes	NR	ctDNA+ vs ctDNA-
Openshaw 2020 (41)	NGS+ddPCR +qPCR	Yes	TP53(82%), ERBB2(11%) , KIF2B(11%), CDKN2A(9%)	ctDNA+ vs ctDNA-
Azad 2020 (42)	NGS(CAPP-Seq)	Both	TP53(84%) , CCND1(24%) , CCNE1(14%) , VEGFA(14%), CDKN2A(14%), PIK3CA(11%), KRAS(11%), MYC(11%), ERBB2(11%)	ctDNA+ vs ctDNA-
Mohamed 2019 (43)	NGS+ddPCR+qPCR	Yes	NR	ctDNA+ vs ctDNA-
Maron 2019 (44)	NGS	Yes	TP53 (53%) , HER2 (17%) , EGFR (17%), KRAS (15%), MYC (13%), PIK3CA (13%) , MET (11%)	ctDNA PIK3CA mutation (Yes vs No)
Kato 2018 (45)	NGS	Yes	TP53 (50.9%), PIK3CA (16.4%), ERBB2 (14.5%), KRAS (14.5%)	ctDNA ERBB2 mutation (Yes vs No)

NR, not reported; NGS, next generation sequencing; dPCR, digital PCR; ddPCR, droplet digital PCR; qPCR, quantitative-PCR; Both, Includes tumor-informed assay and non-tumor-informed assay; VAF, variant allele frequency; ichorCNA: shallow whole-genome sequencing (sWGS)-derived copy number tumor fraction estimates.

### 3.2 ctDNA detection at baseline

**Figure 1:**
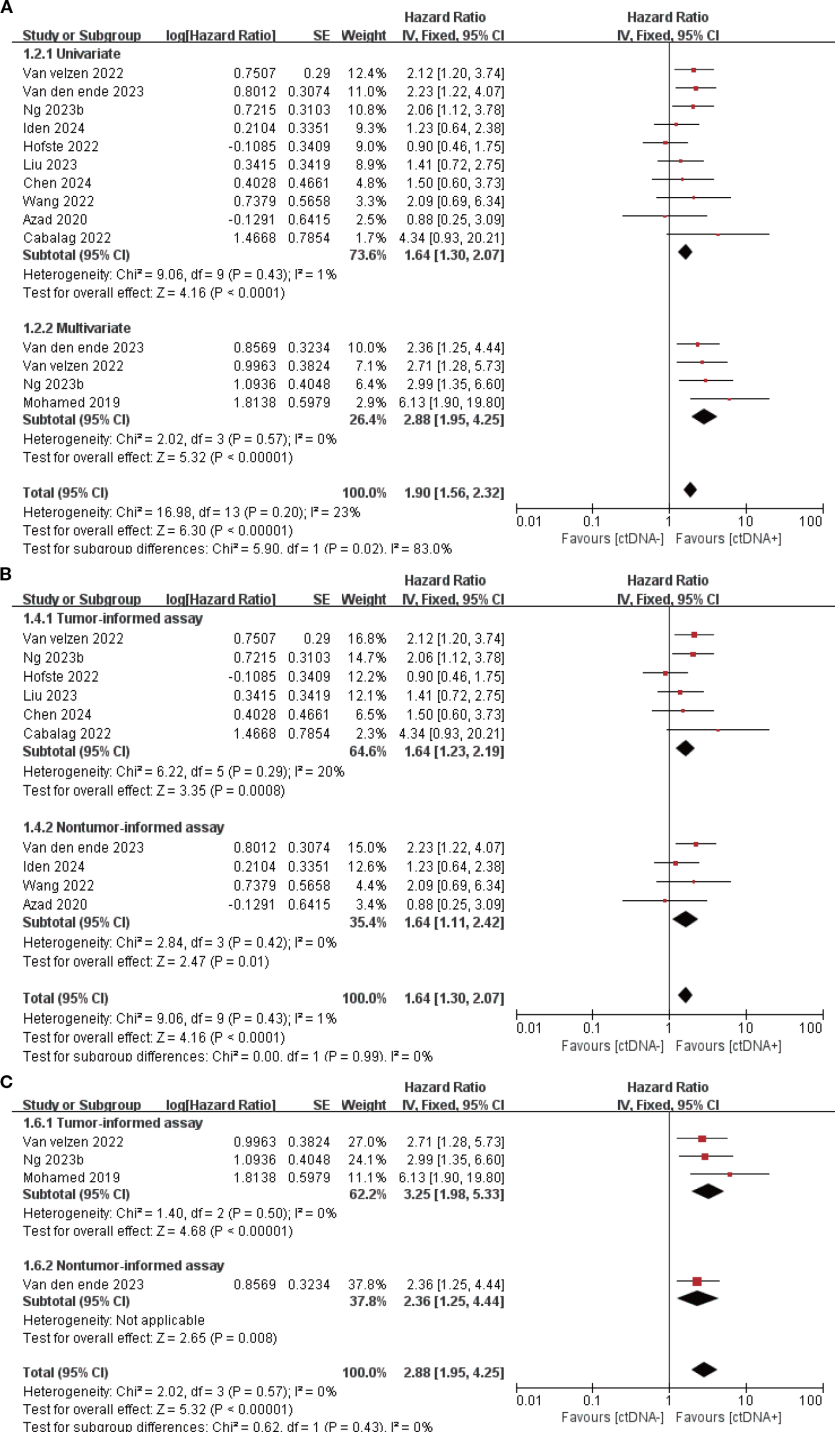
Association of ctDNA testing at baseline with PFS (fixed-effects model). **(A)** Univariate and multivariate analyses. Subgroup analyses of tumor-informed and non-tumor-informed assays in univariate **(B)** and multivariate **(C)** analyses.

**Figure 2:**
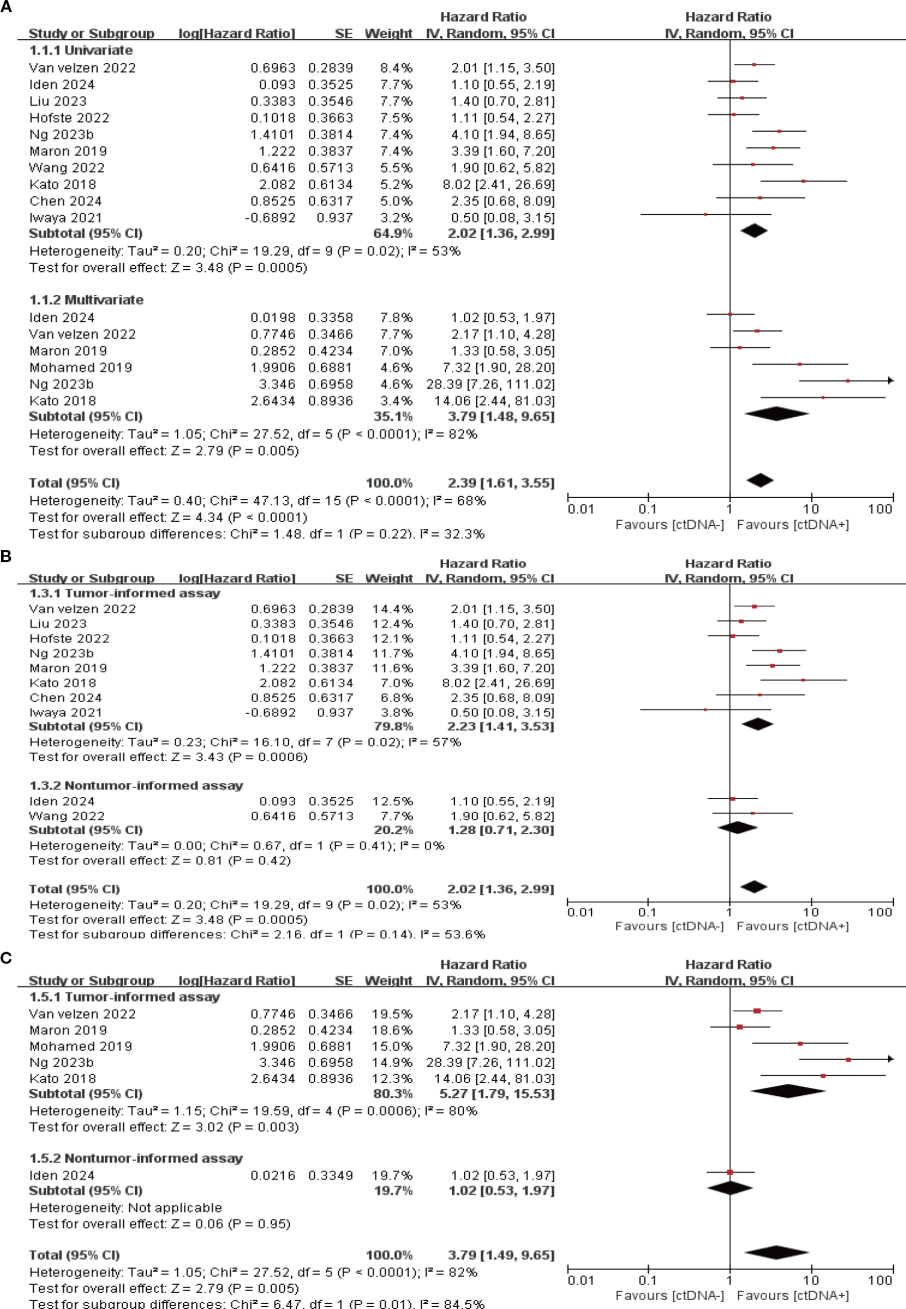
Association of ctDNA testing at baseline with OS (random-effects model). **(A)** Univariate and multivariate analyses. Subgroup analyses of tumor-informed and non-tumor-informed assays in univariate **(B)** and multivariate **(C)** analyses.

11 studies (N = 647 patients) and 11 studies (N = 696 patients) reported associations between ctDNA testing at baseline with PFS and OS, respectively. In univariate and multivariate analyses, a positive ctDNA test at baseline was associated with poorer PFS (univariate: HR = 1.64, 95% CI:1.30-2.07, multivariate: HR = 2.88, 95% CI:1.95-4.25) and OS (univariate: HR = 2.02, 95% CI:1.36-2.99, multivariate: HR = 3.79, 95% CI:1.48-9.65) (**Figure 1A**, **2A**, **Supplementary Table S3**). Similar results were obtained when further subgroup analyses of tumor-informed and non-tumor-informed testing were performed in univariate and multivariate analyses (**Figures 1B, C**, **2B, C**, **Supplementary Table S4**).In addition, the combined HR of studies using tumor-informed testing in univariate and multivariate analyses was numerically higher or equal to that of studies using non-tumor-informed testing (**Figures 1B, C**, **2B, C**, **Supplementary Table S4**). A high degree of statistical heterogeneity could be observed across the full range of analyses in which ctDNA was associated with OS, and we used a random-effects model followed by sensitivity analyses by excluding studies one by one, and found that none of the studies significantly altered the primary outcome. Thus, our results are reliable and stable. **Supplementary Tables S3** and **S4** summarize the associations between ctDNA testing and survival outcomes at different time points.

### 3.3 ctDNA detection after neoadjuvant therapy

**Figure 3:**
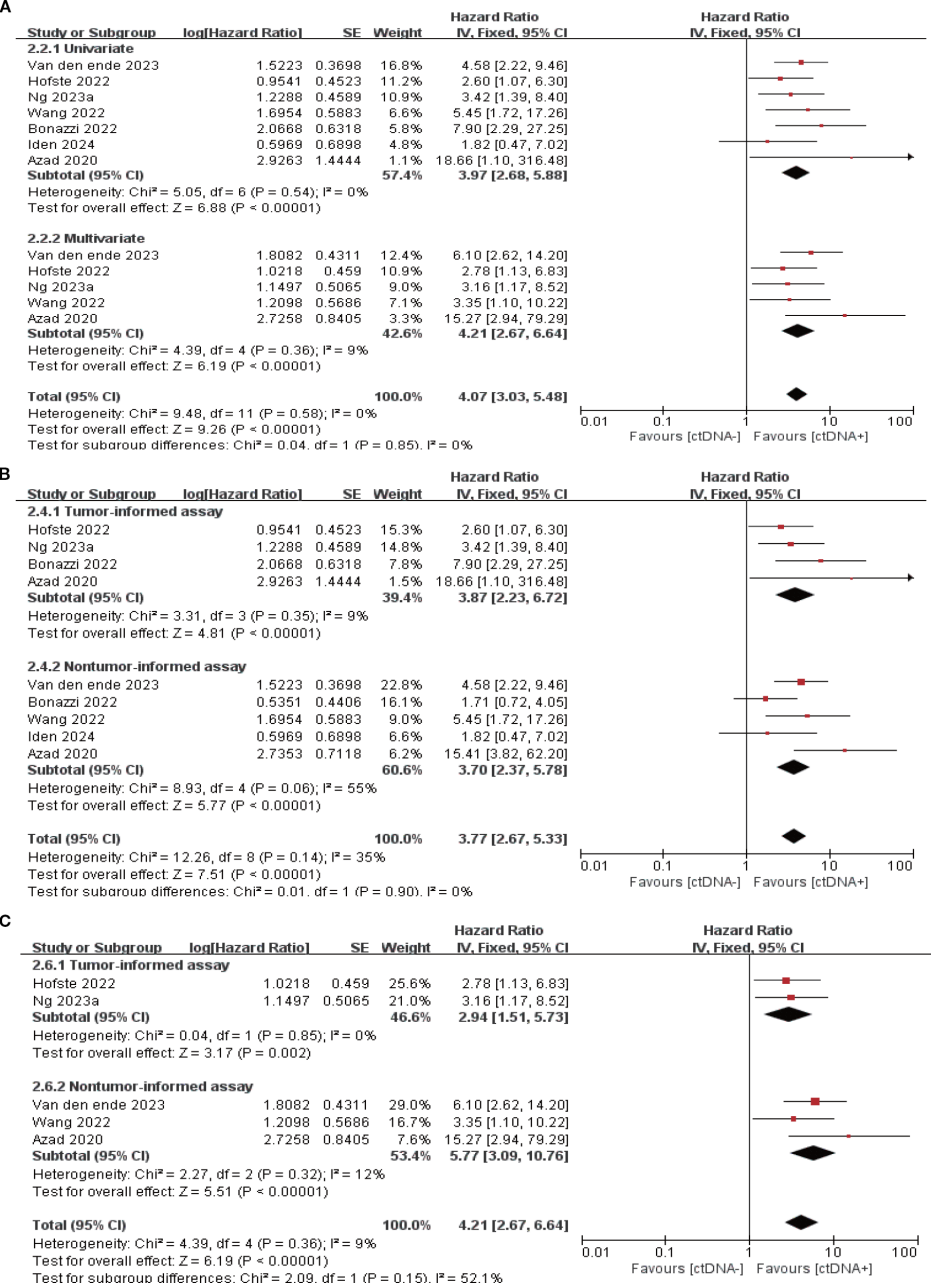
Association of ctDNA testing after neoadjuvant therapy with PFS (fixed-effects model). **(A)** Univariate and multivariate analyses. Subgroup analyses of tumor-informed and non-tumor-informed assays in univariate **(B)** and multivariate **(C)** analyses.

**Figure 4:**
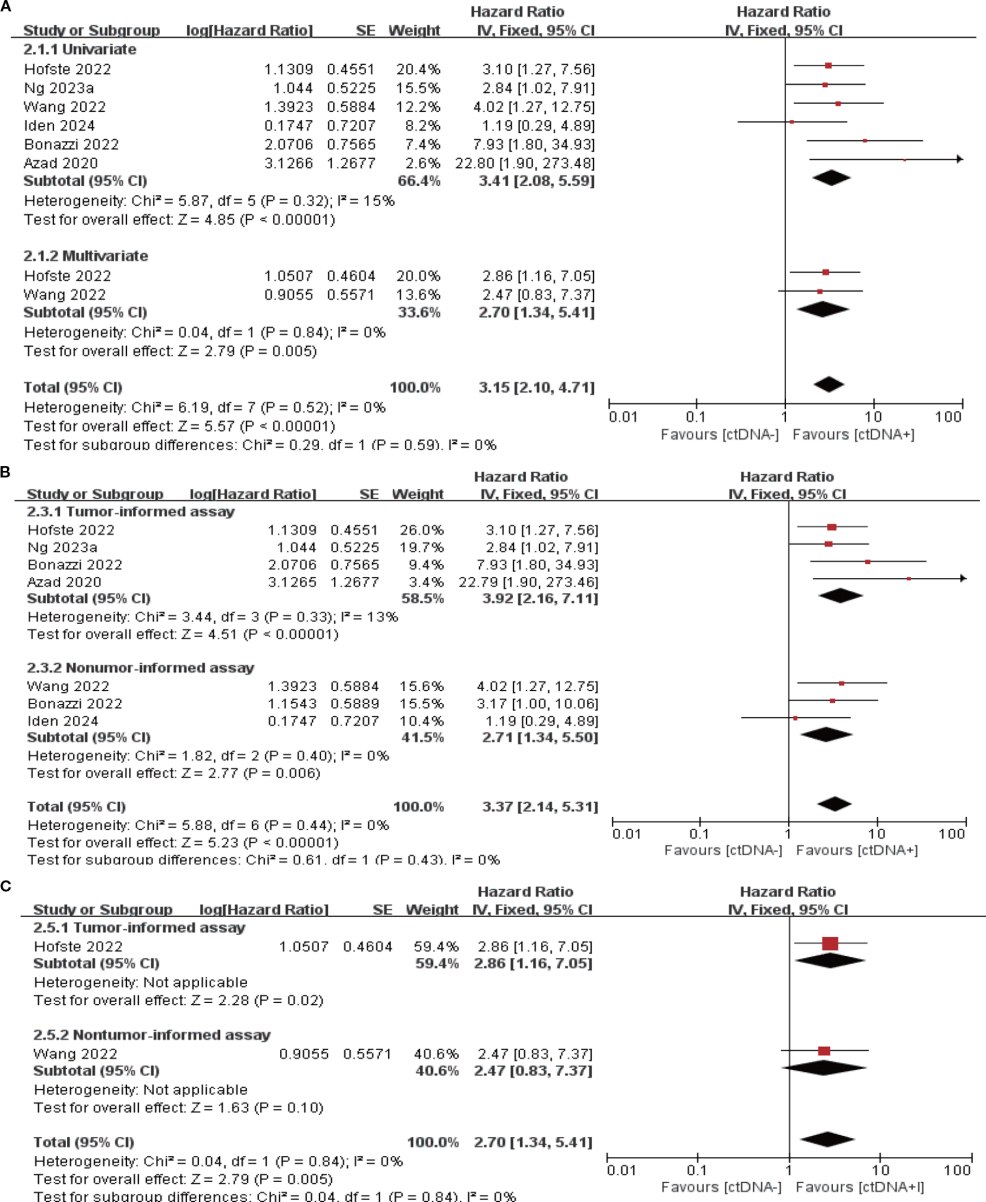
Association of ctDNA testing after neoadjuvant therapy with OS (fixed-effects model). **(A)** Univariate and multivariate analyses. Subgroup analyses of tumor-informed and non-tumor-informed assays in univariate **(B)** and multivariate **(C)** analyses.

7 studies (N = 301 patients) and 6 studies (N = 225 patients) reported the association between ctDNA testing after neoadjuvant therapy with PFS and OS, respectively. In univariate and multivariate analyses, a positive ctDNA test after neoadjuvant therapy was associated with poorer PFS (univariate: HR = 3.97, 95% CI:2.68-5.88, multivariate: HR = 4.21, 95% CI:2.67-6.64) and OS (univariate: HR = 3.41, 95% CI:2.08-5.59, multivariate: HR = 2.70, 95% CI:1.34-5.41) (**Figures 3A**, **4A**, **Supplementary Table S3**). Similar results were obtained when further subgroup analyses of tumor-informed and non-tumor-informed testing were performed in univariate and multivariate analyses (**Figures 3B, C**, **4B, C**, **Supplementary Table S4**). In addition, the combined HR was numerically higher for most of the studies using tumor-informed testing in univariate and multivariate analyses than for non-tumor-informed testing (**Figures 3B**, **4B, C**, **Supplementary Table S4**). In addition, we found that the combined univariate and multivariate HRs for PFS/OS were numerically higher when ctDNA was detected after neoadjuvant therapy(PFS: HR = 4.07, OS: HR = 3.15) than at baseline (PFS: HR = 1.90, OS: HR = 2.39) (**Supplementary Table S3**).

### 3.4 ctDNA detection during the follow-up period

**Figure 5:**
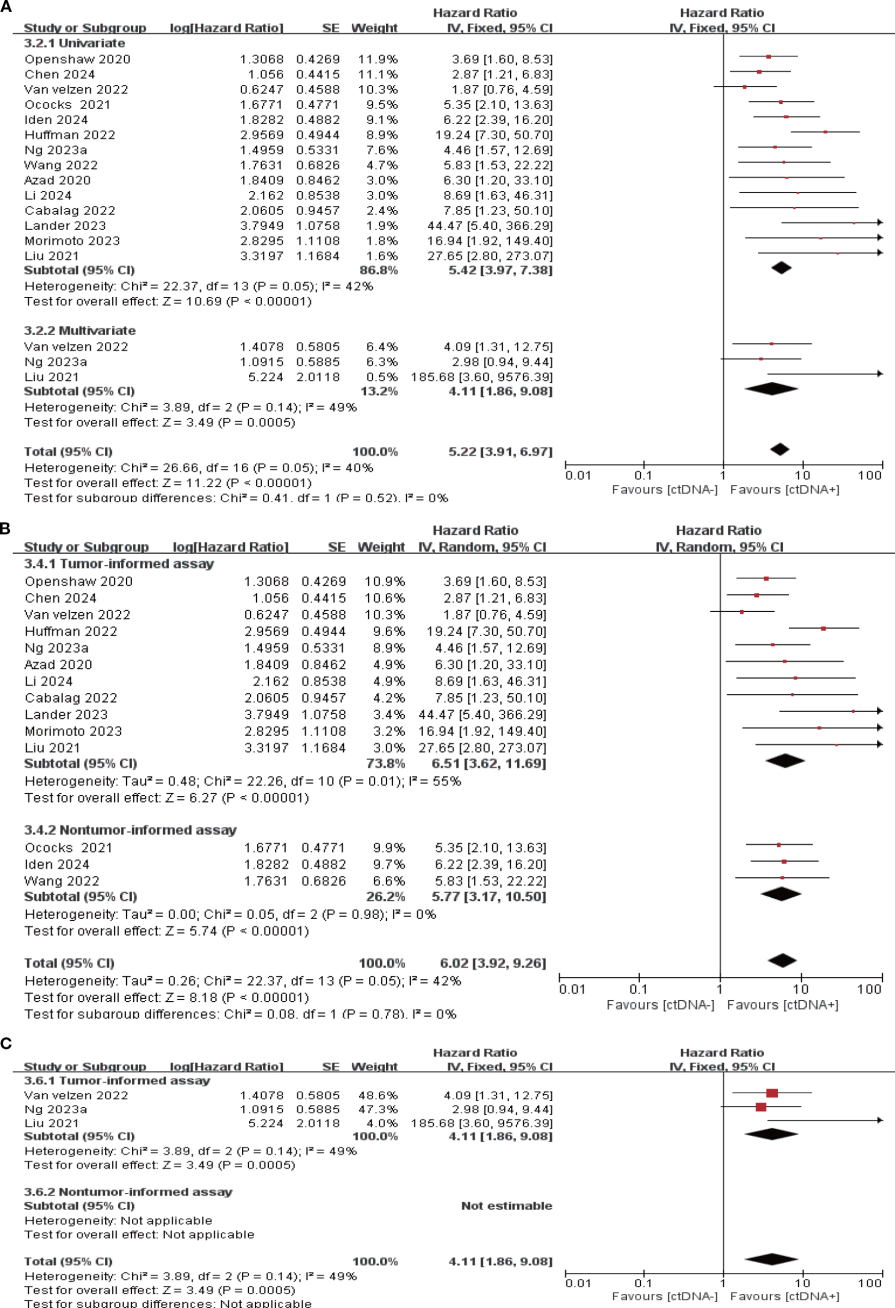
Association of ctDNA testing during follow-up with PFS (fixed/random-effects model). **(A)** Univariate and multivariate analyses. Subgroup analyses of tumor-informed and non-tumor-informed assays in univariate **(B)** and multivariate **(C)** analyses.

**Figure 6:**
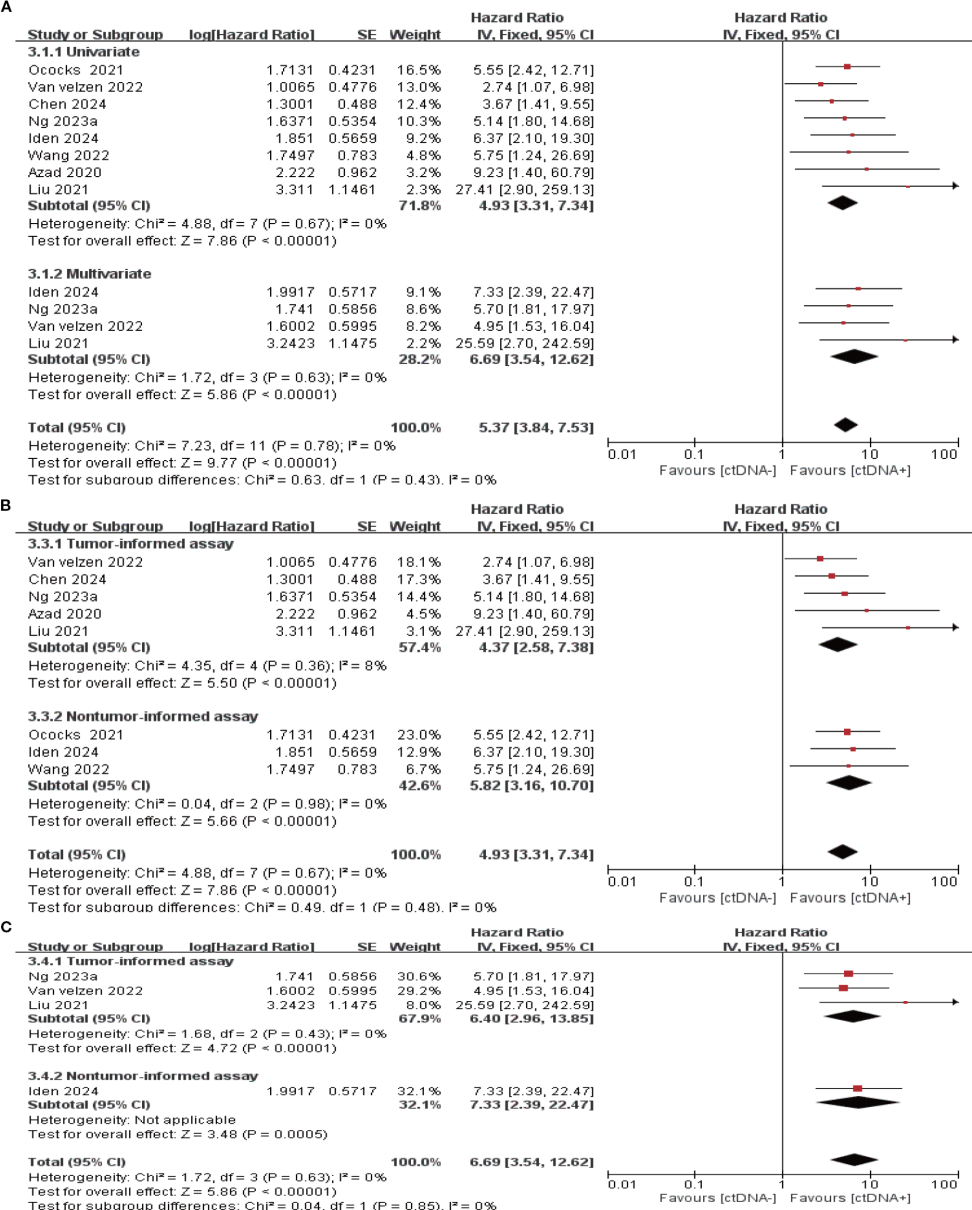
Association of ctDNA testing during follow-up with OS (fixed-effects model). **(A)** Univariate and multivariate analyses. Subgroup analyses of tumor-informed and non-tumor-informed assays in univariate **(B)** and multivariate **(C)** analyses.

14 studies (N = 470 patients) and 8 studies (N = 274 patients) reported the association between ctDNA testing at follow-up with PFS and OS, respectively. In univariate and multivariate analyses, a positive ctDNA test at follow-up was associated with poorer PFS (univariate: HR = 5.42, 95% CI:3.97-7.38, multivariate: HR = 4.11, 95% CI:1.86-9.08) and OS (univariate: HR = 4.93, 95% CI:3.31-7.34, multivariate: HR = 6.69, 95% CI:3.54-12.62) (**Figure 5A**, **6A**, **Supplementary Table S3**). Similar results were obtained when further subgroup analyses of tumor-informed and non-tumor-informed testing were performed in univariate and multivariate analyses (**Figures 5B, C**, **6B, C**, **Supplementary Table S4**). In addition, we found that the combined univariate and multivariate HRs for PFS/OS were numerically higher when ctDNA was detected during follow-up (PFS: HR = 5.22, OS: HR = 5.37) than after neoadjuvant therapy (PFS: HR = 4.07, OS: HR = 3.15) and at baseline (PFS: HR = 1.90, OS: HR = 2.39) (**Supplementary Table S3**).

### 3.5 Lead time

There are 9 studies (26, 28, 29, 32, 36, 40-43) comparing the mean time to the prediction of clinical recurrence by ctDNA testing with conventional radiological imaging techniques (e.g., CT, PET-CT). The results showed that ctDNA test positivity predicted clinical recurrence on average 4.53 months earlier (range: 0.98-11.6 months) than imaging techniques.

### 3.6 Risk of bias assessment and publication bias

We used the Newcastle-Ottawa scale to assess the risk of bias for the included studies. The results showed that all 22 included studies scored≥6. **Supplementary Table S5** comprehensively describes the results of the quality assessment of the included literature. Publication bias was assessed using a funnel plot for the univariate analysis of baseline PFS, baseline OS, and follow-up PFS (**Supplementary Figure S2**). The results suggest the possibility of publication bias in the univariate analysis of follow-up PFS.

## 4 Discussion

This study differs from previous systematic reviews and meta-analyses in that it systematically elucidates the prognostic value of ctDNA in EC from a dynamic temporal perspective. By employing univariate and multivariate statistical methods combined with tumor-informed and non-informed analysis strategies, we provide a more comprehensive, time-specific basis for risk stratification and efficacy assessment.

Genomic alterations are key drivers of EC development and metastasis (46). Studies indicate that the most frequently mutated genes in ESCC include TP53, NFE2L2, and MLL2, while EAC predominantly involves TP53, CDKN2A, and ARID1A (47). Among these, TP53 exhibits the highest mutation rate in both subtypes (47–49) and correlates with poorer survival outcomes (50). Since Hsieh et al. (51) first reported the association between plasma cell-free DNA and EC recurrence and survival, numerous prospective and retrospective studies have focused on the prognostic value of ctDNA in EC. This systematic review and meta-analysis primarily assessed the clinical significance of ctDNA monitoring at three time points: baseline, after neoadjuvant therapy, and during follow-up.

Baseline ctDNA positivity is often associated with greater tumor burden and more advanced clinical staging (25–27, 33, 35, 43), suggesting its potential as a biomarker reflecting EC tumor burden and assessing prognosis. Although some included studies did not find a significant association with prognosis (25, 26, 32, 35, 40), others reported a clear correlation (27, 28, 33, 36, 43). The pooled results of this meta-analysis indicate that baseline ctDNA positivity is a significant risk factor for recurrence and mortality in EC patients, consistent with reports in other cancer types such as breast and lung cancer (15, 52, 53). Further analysis revealed that ctDNA positivity detected after neoadjuvant therapy and during follow-up was also significantly associated with higher risks of recurrence and mortality. Notably, the combined hazard ratio (HR) for recurrence or death following ctDNA positivity after neoadjuvant therapy was approximately 1–2 times that of baseline status, suggesting superior predictive efficacy. The HR for ctDNA positivity during follow-up further increased to 1–2 times that after neoadjuvant therapy, indicating that its prognostic value progressively escalates over time, peaking during follow-up. This precisely quantified pattern of risk evolution, systematically elucidated for the first time in the EC field through meta-analysis, highlights the significant advantage of dynamic monitoring over single-time-point detection. Notably, this pattern of risk change highly correlates with the dynamic clearance pattern of ctDNA. Multiple studies consistently demonstrate that a decrease or clearance of ctDNA levels during or after treatment is significantly associated with improved patient survival, with those maintaining persistent negativity or achieving clearance exhibiting better prognosis (25, 26, 32). Therefore, our discovery of this dynamic pattern represents a significant addition to previous review conclusions and provides a solid foundation for the clinical translation of ctDNA.

More importantly, viewed from the perspective of tumor ecology and evolution (54), these dynamic patterns reflect the adaptive evolution of tumor clonal populations under therapeutic pressure. Persistent ctDNA positivity after neoadjuvant therapy may indicate the selection and amplification of adaptive resistance subclones, while its clearance may reflect effective suppression of the dominant clone by treatment. This understanding extends beyond the static interpretation framework of traditional molecular biomarkers, situating ctDNA dynamics within the ecological system of tumor-microenvironment-therapy interactions. It provides a novel theoretical perspective for deepening our understanding of treatment response and resistance mechanisms.

Based on these time-specific risk quantification evidence and theoretical understanding, persistent ctDNA positivity after neoadjuvant therapy should be regarded as a strong indicator of tumor resistance to treatment and extremely high recurrence risk. Regardless of imaging findings, these patients should be considered for intensified adjuvant therapy (e.g., immunotherapy, extended chemotherapy, or targeted therapy) to eradicate residual disease. For ctDNA positivity emerging during follow-up, immediate initiation of more frequent imaging (e.g., PET-CT) is warranted, along with exploring preemptive treatment before clinical recurrence. This study provides evidence-based support for incorporating ctDNA-guided temporal stratification into the comprehensive management of EC patients, advancing from prognostic assessment to personalized intervention. However, the primary challenge to clinical translation remains the absence of standardized detection thresholds and unified reporting standards. Future research should focus on establishing clinically actionable criteria for ctDNA positivity at various time points. Prospective intervention trials—such as randomized controlled trials evaluating intensified treatment strategies for ctDNA-positive patients—are needed to validate the actual survival benefit. Ongoing clinical trials (e.g., NCT06914011, NCT05965479) provide an initial framework for ctDNA-guided therapy.

In colorectal and breast cancers, tumor-informed ctDNA testing demonstrated superior performance compared to non-tumor-informed assay, exhibiting higher sensitivity (52, 55, 56). This study found that the prognostic value of ctDNA derived from both testing methods was generally consistent across three time points: baseline, after neoadjuvant therapy, and follow-up. Notably, at baseline and after neoadjuvant therapy stages, the hazard ratios (HRs) for recurrence and mortality risk derived from the tumor-informed assay tended to be numerically higher than those from the non-tumor-informed assay, suggesting potential advantages during periods of high tumor burden due to greater sensitivity. However, given the limited research data and high uncertainty surrounding the non-tumor-informed assay, the actual effect of the tumor-informed assay may be overestimated. The conclusion presented here is only a preliminary hypothesis: a tumor-informed assay may be more sensitive in identifying high-risk recurrence and mortality in EC patients. Currently, systematic comparative studies in the EC field are lacking, and further research is needed to clarify the differences and clinical applicability of these two strategies.

During the tumor-free interval following treatment, when imaging fails to detect lesions, serial liquid biopsies can be used for early prediction of recurrence risk. This study found that in EC, the emergence of ctDNA positivity occurred on average 4.53 months earlier than imaging detection. This trend has been consistently reported across multiple tumor types, including breast cancer and colorectal cancer (52, 57, 58). Current clinical guidelines still regard imaging as the gold standard for recurrence diagnosis and treatment decisions. It is important to note that intervention based solely on ctDNA positivity carries risks, such as false positives due to clonal hematopoiesis or technical errors, which may lead to overtreatment. Therefore, after detecting ctDNA positivity, it is recommended to promptly combine imaging examinations for careful evaluation to ensure robust clinical decision-making and patient safety. Furthermore, the current early warning value of ctDNA remains based on limited studies and requires further validation across diverse populations and healthcare settings. Future large-scale, rigorously designed multicenter prospective studies are necessary to confirm these findings.

This study has several limitations. First, outcome measures were grouped into two broad categories—”recurrence-related (including PFS, DFS, RFS, and FFP)” and “mortality-related (including OS, DSS, and CSD)”—resulting in a certain lack of outcome consistency. Second, criteria for defining ctDNA positivity varied across studies, and the absence of standardized testing protocols reduced comparability of results. Third, the study did not perform stratified analyses by histological subtype (EAC vs. ESCC) or geographic region (Asia vs. Western countries). Given the significant molecular and clinical differences between these subtypes, along with regional heterogeneity in clinical practice and epidemiological backgrounds, this limitation restricts the generalizability of findings and the interpretability of subgroup analyses. Additionally, substantial heterogeneity existed in baseline OS analyses. Although random-effects models and sensitivity analyses were employed to ensure robustness, interpretation of results may still be affected. At the same time, the studies included primarily utilized univariate HRs, which may introduce residual confounding bias. This heterogeneity and bias primarily stem from clinical and methodological variations. Moreover, the included studies were evenly split between prospective and retrospective designs. Although quality assessments controlled for bias, further high-quality prospective studies are needed for validation. Finally, despite comprehensive search strategies, publication bias may still exist in some analyses. Given these limitations, future research should focus on: ① Establishing unified standards for ctDNA detection and reporting to enhance comparability of results; ② Conducting large-scale, multicenter, prospective studies targeting specific histological subtypes and regional populations, and integrating multidimensional data for individual patient data (IPD)-based meta-analyses to elucidate interactions among factors and more accurately assess the prognostic value of ctDNA; ③ Collectively enhancing the reliability and clinical applicability of relevant evidence through the aforementioned measures.

## 5 Conclusion

Throughout the entire treatment course, positive ctDNA detection consistently correlated with poor prognosis in EC patients. Notably, its prognostic value increased over time, continuously strengthening from baseline to follow-up stages. This study supports ctDNA as an effective dynamic prognostic biomarker and preliminarily explores its potential feasibility for integration into the treatment management workflow for EC patients.measures

## Data Availability

The original contributions presented in the study are included in the article/**Supplementary Material**. Further inquiries can be directed to the corresponding author/s.
