# Supplementary material for: Circulating tumor DNA predicts prognosis at different time points in patients with esophageal cancer: a systematic review and meta-analysis
Source: Front Oncol. 2025 Sep 25;15:1608872. doi: 10.3389/fonc.2025.1608872 (PMC12507613; doi:10.3389/fonc.2025.1608872)
Supplement: Supplementary file 1 [file DataSheet1.pdf]

Supplementary Material

1 Supplementary Figures and Tables

1.1 Supplementary Figures

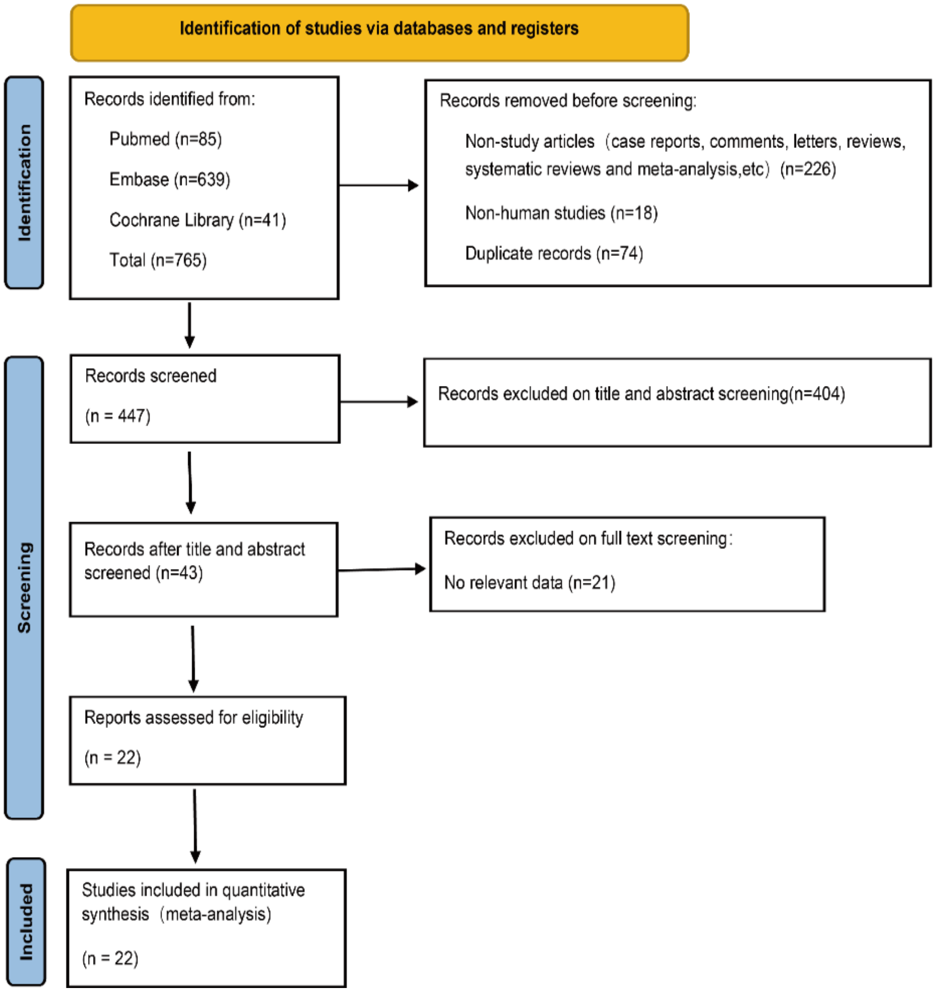

Supplementary Figure S1. Literature search and study selection according to PRISMA 2020 flow diagram for systematic review.

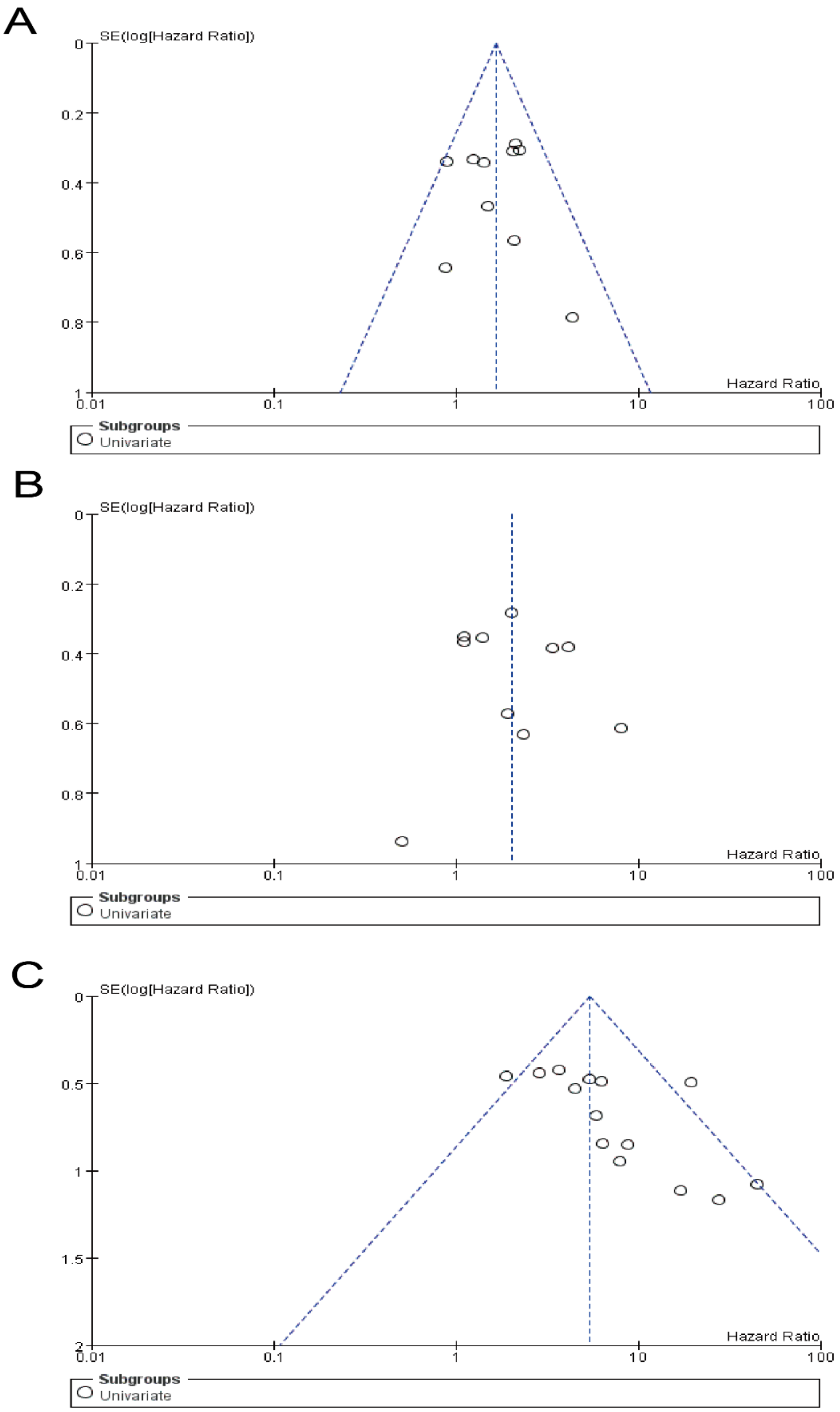

Supplementary Figure S2. Publication bias assessment. A. Univariate analysis of PFS at baseline; B. Univariate analysis of OS at baseline; C. Univariate analysis of PFS during follow-up.

1.2 Supplementary Tables

Supplementary Table S1. PRISMA 2020 Checklist.

Section and Topic	Item #	Checklist item	Location where item is reported
**TITLE**			1
Title	1	Identify the report as a systematic review.	
**ABSTRACT**			1-2
Abstract	2	See the PRISMA 2020 for Abstracts checklist.	
**INTRODUCTION**			2
Rationale	3	Describe the rationale for the review in the context of existing knowledge.	
Objectives	4	Provide an explicit statement of the objective(s) or question(s) the review addresses.	
**METHODS**			2-3
Eligibility criteria	5	Specify the inclusion and exclusion criteria for the review and how studies were grouped for the syntheses.	
Information sources	6	Specify all databases, registers, websites, organisations, reference lists and other sources searched or consulted to identify studies. Specify the date when each source was last searched or consulted.	
Search strategy	7	Present the full search strategies for all databases, registers and websites, including any filters and limits used.	
Selection process	8	Specify the methods used to decide whether a study met the inclusion criteria of the review, including how many reviewers screened each record and each report retrieved, whether they worked independently, and if applicable, details of automation tools used in the process.	
Data collection process	9	Specify the methods used to collect data from reports, including how many reviewers collected data from each report, whether they worked independently, any processes for obtaining or confirming data from study investigators, and if applicable, details of automation tools used in the process.	
Data items	10a	List and define all outcomes for which data were sought. Specify whether all results that were compatible with each outcome domain in each study were sought (e.g. for all measures, time points, analyses), and if not, the methods used to decide which results to collect.	
	10b	List and define all other variables for which data were sought (e.g. participant and intervention characteristics, funding sources). Describe any assumptions made about any missing or unclear information.	
Study risk of bias assessment	11	Specify the methods used to assess risk of bias in the included studies, including details of the tool(s) used, how many reviewers assessed each study and whether they worked independently, and if applicable, details of automation tools used in the process.	
Effect measures	12	Specify for each outcome the effect measure(s) (e.g. risk ratio, mean difference) used in the synthesis or presentation of results.	
Synthesis methods	13a	Describe the processes used to decide which studies were eligible for each synthesis (e.g. tabulating the study intervention characteristics and comparing against the planned groups for each synthesis (item #5)).	
	13b	Describe any methods required to prepare the data for presentation or synthesis, such as handling of missing summary statistics, or data conversions.	
	13c	Describe any methods used to tabulate or visually display results of individual studies and syntheses.	
	13d	Describe any methods used to synthesize results and provide a rationale for the choice(s). If meta-analysis was performed, describe the model(s), method(s) to identify the presence and extent of statistical heterogeneity, and software package(s) used.	
	13e	Describe any methods used to explore possible causes of heterogeneity among study results (e.g. subgroup analysis, meta-regression).	
	13f	Describe any sensitivity analyses conducted to assess robustness of the synthesized results.	
Reporting bias assessment	14	Describe any methods used to assess risk of bias due to missing results in a synthesis (arising from reporting biases).	
Certainty assessment	15	Describe any methods used to assess certainty (or confidence) in the body of evidence for an outcome.	
**RESULTS**			3-10
Study selection	16a	Describe the results of the search and selection process, from the number of records identified in the search to the number of studies included in the review, ideally using a flow diagram.	
	16b	Cite studies that might appear to meet the inclusion criteria, but which were excluded, and explain why they were excluded.	
Study characteristics	17	Cite each included study and present its characteristics.	
Risk of bias in studies	18	Present assessments of risk of bias for each included study.	
Results of individual studies	19	For all outcomes, present, for each study: (a) summary statistics for each group (where appropriate) and (b) an effect estimate and its precision (e.g. confidence/credible interval), ideally using structured tables or plots.	
Results of syntheses	20a	For each synthesis, briefly summarise the characteristics and risk of bias among contributing studies.	
	20b	Present results of all statistical syntheses conducted. If meta-analysis was done, present for each the summary estimate and its precision (e.g. confidence/credible interval) and measures of statistical heterogeneity. If comparing groups, describe the direction of the effect.	
	20c	Present results of all investigations of possible causes of heterogeneity among study results.	
	20d	Present results of all sensitivity analyses conducted to assess the robustness of the synthesized results.	

Reporting biases	21	Present assessments of risk of bias due to missing results (arising from reporting biases) for each synthesis assessed.	
Certainty of evidence	22	Present assessments of certainty (or confidence) in the body of evidence for each outcome assessed.	
**DISCUSSION**			11-14
Discussion	23a	Provide a general interpretation of the results in the context of other evidence.	
	23b	Discuss any limitations of the evidence included in the review.	
	23c	Discuss any limitations of the review processes used.	
	23d	Discuss implications of the results for practice, policy, and future research.	
**OTHER INFORMATION**			
Registration and protocol	24a	Provide registration information for the review, including register name and registration number, or state that the review was not registered.	2
	24b	Indicate where the review protocol can be accessed, or state that a protocol was not prepared.	2
	24c	Describe and explain any amendments to information provided at registration or in the protocol.	NA
Support	25	Describe sources of financial or non-financial support for the review, and the role of the funders or sponsors in the review.	14
Competing interests	26	Declare any competing interests of review authors.	14
Availability of data, code and other materials	27	Report which of the following are publicly available and where they can be found: template data collection forms; data extracted from included studies; data used for all analyses; analytic code; any other materials used in the review.	14

From: Page MJ, McKenzie JE, Bossuyt PM, Boutron I, Hoffmann TC, Mulrow CD, et al. The PRISMA 2020 statement: an updated guideline for reporting systematic reviews. BMJ 2021;372:n71. doi: 10.1136/bmj.n71. This work is licensed under CC BY 4.0. To view a copy of this license, visit https://creativecommons.org/licenses/by/4.0/

Supplementary Table S2. search strategy.

Search step	Query	Results
	PubMed	
**#1**	"Circulating Tumor DNA"[Mesh]	3,125
**#2**	((((((Circulating Tumor DNA[Title/Abstract]) OR (DNA, Circulating Tumor[Title/Abstract])) OR (Tumor DNA, Circulating[Title/Abstract])) OR (Cell-Free Tumor DNA[Title/Abstract])) OR (Cell Free Tumor DNA[Title/Abstract])) OR (DNA, Cell-Free Tumor[Title/Abstract])) OR (Tumor DNA, Cell-Free[Title/Abstract])	5,228
**#3**	#1 OR #2	6,182
**#4**	"Esophageal Neoplasms"[Mesh]	62,025

**#5**	((((((((((((((((Esophageal Neoplasms[Title/Abstract]) OR (Esophageal Neoplasm[Title/Abstract])) OR (Neoplasm, Esophageal[Title/Abstract])) OR (Esophagus Neoplasm[Title/Abstract])) OR (Esophagus Neoplasms[Title/Abstract])) OR (Neoplasm, Esophagus[Title/Abstract])) OR (Neoplasms, Esophagus[Title/Abstract])) OR (Neoplasms, Esophageal[Title/Abstract])) OR (Cancer of Esophagus[Title/Abstract])) OR (Cancer of the Esophagus[Title/Abstract])) OR (Esophagus Cancer[Title/Abstract])) OR (Cancer, Esophagus[Title/Abstract])) OR (Cancers, Esophagus[Title/Abstract])) OR (Esophagus Cancers[Title/Abstract])) OR (Esophageal Cancer[Title/Abstract])) OR (Cancer, Esophageal[Title/Abstract])) OR (Cancers, Esophageal[Title/Abstract])) OR (Esophageal Cancers[Title/Abstract])	32,509
**#6**	#4 OR #5	70,874
**#7**	#3 AND #6	85
	Embase	
**#1**	'circulating tumor dna'/exp	12045
**#2**	'circulating tumor dna'/exp OR 'circulating tumor dna' OR (circulating AND ('tumor'/exp OR tumor) AND ('dna'/exp OR dna)) OR 'cell-free circulating tumor dna':ab,ti OR 'cell-free circulating tumour dna':ab,ti OR 'cell-free tumor dna':ab,ti OR 'cell-free tumour dna':ab,ti OR 'circulating cell-free tumor dna':ab,ti OR 'circulating cell-free tumour dna':ab,ti OR 'circulating tumour dna':ab,ti OR 'circulating tumor dna':ab,ti	30040
**#3**	#1 OR #2	30040
**#4**	'esophagus tumor'/exp	117361
**#5**	'esophagus tumor'/exp OR 'esophagus tumor' OR (('esophagus'/exp OR esophagus) AND ('tumor'/exp OR tumor)) OR ('esophageal mass':ab,ti AND tumor:ab,ti) OR ('esophageal masses':ab,ti AND tumor:ab,ti) OR 'esophageal neoplasms':ab,ti OR 'esophageal tumor':ab,ti OR 'esophageal tumorigenesis':ab,ti OR 'esophagus neoplasm':ab,ti OR 'esophagus tumour':ab,ti OR 'neoplasia of the esophagus':ab,ti OR 'neoplasia of the oesophagus':ab,ti OR 'neoplasm of the esophagus':ab,ti OR 'neoplastic esophageal':ab,ti OR 'neoplastic esophagus':ab,ti OR 'neoplastic oesophageal':ab,ti OR 'oesophageal neoplasms':ab,ti OR 'oesophageal tumor':ab,ti OR 'oesophageal tumour':ab,ti OR 'oesophagus tumor':ab,ti OR 'oesophagus tumour':ab,ti OR 'tumor of the esophagus':ab,ti OR 'tumor, esophagus':ab,ti OR 'tumors of the esophagus':ab,ti OR 'tumour of the oesophagus':ab,ti OR 'tumour, esophagus':ab,ti OR 'tumours of the oesophagus':ab,ti OR 'esophagus tumor':ab,ti	163215
**#6**	#4 OR #5	163215
**#7**	#3 AND #6	639
	Cochrane	

**#1**	MeSH descriptor: [Circulating Tumor DNA] explode all trees	100
**#2**	(Circulating Tumor DNA):ti,ab,kw OR (DNA, Circulating Tumor):ti,ab,kw OR (Tumor DNA, Circulating):ti,ab,kw OR (Cell-Free Tumor DNA):ti,ab,kw OR (Cell Free Tumor DNA):ti,ab,kw	1969
**#3**	(Circulating Tumor DNA):ti,ab,kw OR (Tumor DNA, Cell-Free):ti,ab,kw OR (DNA, Cell-Free Tumor):ti,ab,kw	1336
**#4**	#1 OR #2 OR #3	1969
**#5**	MeSH descriptor: [Esophageal Neoplasms] explode all trees	2641
**#6**	(Esophageal Neoplasms):ti,ab,kw OR (Neoplasms, Esophageal):ti,ab,kw OR (Esophagus Neoplasms):ti,ab,kw OR (Neoplasms, Esophagus):ti,ab,kw OR (Esophagus Neoplasm):ti,ab,kw	3591
**#7**	(Neoplasm, Esophageal):ti,ab,kw OR (Neoplasm, Esophageal):ti,ab,kw OR (Esophageal Neoplasm):ti,ab,kw OR (Esophagus Cancers):ti,ab,kw OR (Cancer, Esophagus):ti,ab,kw	3689
**#8**	(Cancer of the Esophagus):ti,ab,kw OR (Cancer of Esophagus):ti,ab,kw OR (Esophageal Cancers):ti,ab,kw OR (Cancers, Esophageal):ti,ab,kw OR (Esophageal Cancer):ti,ab,kw	6082
**#9**	(Esophagus Cancer):ti,ab,kw OR (Cancer, Esophageal):ti,ab,kw OR (Cancers, Esophagus):ti,ab,kw	6079
**#10**	#5 OR #6 OR #7 OR #8 OR #9	6988
**#11**	#4 AND #10	41

Supplementary Table S3. Univariate and multivariate analyses of ctDNA testing and outcome (PFS/OS) at different time points.

Timepoint	Endpoint		Studies	Participants	HR (95% CI)
Baseline	PFS	Univariate	10	614	1.64 (1.30, 2.07)
		Multivariate	4	246	2.88 (1.95, 4.25)
		Overall	11	647	1.90 (1.56, 2.32)
	OS	Univariate	10	651	2.02 (1.36, 2.99)
		Multivariate	6	447	3.79 (1.48, 9.65)
		Overall	11	696	2.39 (1.61, 3.55)
After neoadjuvant therapy	PFS	Univariate	7	295	3.97 (2.68, 5.88)
		Multivariate	5	217	4.21 (2.67, 6.64)
		Overall	7	301	4.07 (3.03, 5.48)
	OS	Univariate	6	219	3.41 (2.08, 5.59)
		Multivariate	2	77	2.70 (1.34, 5.41)
		Overall	6	225	3.15 (2.10, 4.71)
Follow-up period	PFS	Univariate	14	470	5.42 (3.97, 7.38)
		Multivariate	3	85	4.11 (1.86, 9.08)
		Overall	14	470	5.22 (3.91, 6.97)
	OS	Univariate	8	274	4.93 (3.31, 7.34)
		Multivariate	4	140	6.69 (3.54, 12.62)

		Overall	8	274	5.37 (3.84, 7.53)

Supplementary Table S4. Subgroup analysis of tumour-informed versus non-tumour-informed assays in univariate and multivariate analyses of ctDNA testing and outcome (PFS/OS) at different time points.

**Timepoint**	**Endpoint**		**Studies**	**Tumor informed**	**Nontumor-informed**
Baseline	PFS	Univariate	10	1.64 (1.23, 2.19)	1.64 (1.11, 2.42)
		Multivariate	4	3.25 (1.98, 5.33)	2.36 (1.25, 4.44)
	OS	Univariate	10	2.23 (1.41, 3.53)	1.28 (0.71, 2.30)
		Multivariate	6	5.27 (1.79, 15.53)	1.02 (0.53, 1.97)
After neoadjuvant therapy	PFS	Univariate	7	3.87 (2.23, 6.72)	3.70 (2.37, 5.78)
		Multivariate	5	2.94 (1.51, 5.73)	5.77 (3.09,10.76)
	OS	Univariate	6	3.92 (2.16, 7.11)	2.71 (1.34, 5.50)
		Multivariate	2	2.86 (1.16, 7.05)	2.47 (0.83, 7.37)
Follow-up period	PFS	Univariate	14	6.51 (3.62, 11.69)	5.77 (3.17, 10.50)
		Multivariate	3	4.11 (1.86, 9.08)	—
	OS	Univariate	8	4.37 (2.58, 7.38)	5.82 (3.16, 10.70)
		Multivariate	4	6.40 (2.96, 13.85)	7.33 (2.39, 22.47)

Supplementary table S5 . The assessment of the risk of bias included studies using the Newcastle–Ottawa scale.

Study	Selection (0–4)				Comparability (0-2)		Outcome (0–3)			
	**REC**	**SNEC**	**AE**	**DO**	**SC**	**AF**	**AO**	**FU**	**AFU**	**Total**
Li 2024	1	1	1	1	0	0	1	0	1	6
Iden 2024	1	1	1	1	1	0	1	0	1	7
Chen 2024	1	1	1	1	1	0	1	0	1	7
Van den ende 2023	1	1	1	1	1	0	1	1	1	8
Ng 2023a	1	1	1	1	1	0	1	1	1	8
Ng 2023b	1	1	1	1	1	0	1	1	1	8
Morimoto 2023	1	1	1	1	1	0	1	0	1	7
Liu 2023	1	1	1	1	1	0	1	1	1	8
Lander 2023	1	1	1	1	0	0	1	0	1	6
Wang 2022	1	1	1	1	1	0	1	0	1	7
Van velzen 2022	1	1	1	1	1	0	1	1	1	8
Huffman 2022	1	1	1	1	0	0	1	1	1	7
Hofste 2022	1	1	1	1	1	0	1	1	1	8
Cabalag 2022	1	1	1	1	1	0	1	1	1	8
Bonazzi 2022	1	1	1	1	1	0	1	1	1	8
Ococks 2021	1	1	1	1	1	0	1	1	1	8
Liu 2021	1	1	1	1	1	0	1	1	1	8
Iwaya 2021	1	1	1	1	0	0	1	1	1	7
Openshaw 2020	1	1	1	1	0	0	1	1	1	7
Azad 2020	1	1	1	1	1	0	1	1	1	8
Mohamed 2019	1	1	1	1	0	0	1	0	1	6
Maron 2019	1	1	1	1	0	0	1	1	1	7
Kato 2018	1	1	1	1	1	0	1	0	1	7

REC: Representativeness of the exposed cohort; SNEC: Selection of the non-exposed cohort; AE: Ascertainment of exposure; DO: Demonstration that outcome of interest was not present at the start of the study; SC: control for important factors; AF: Study controls for other factors; AO: Assessment of outcome; FU: Follow-up long enough (>36m) for outcomes to occur. AFU: Adequacy of follow-up of cohorts(＞80%).
